# Role of body size and shape in animal camouflage

**DOI:** 10.1002/ece3.11434

**Published:** 2024-05-13

**Authors:** Hongmin Yu, Zhixue Lin, Fanrong Xiao

**Affiliations:** ^1^ Ministry of Education Key Laboratory for Ecology of Tropical Islands, Key Laboratory of Tropical Animal and Plant Ecology of Hainan Province, College of Life Sciences Hainan Normal University Haikou China

**Keywords:** anti‐predation, crypsis, masquerade camouflage, morphology

## Abstract

Animal camouflage serves a dual purpose in that it enhances both predation efficiency and anti‐predation strategies, such as background matching, disruptive coloration, countershading, and masquerade, for predators and prey, respectively. Although body size and shape determine the appearance of animals, potentially affecting their camouflage effectiveness, research over the past two centuries has primarily focused on animal coloration. Over the past two decades, attention has gradually shifted to the impact of body size and shape on camouflage. In this review, we discuss the impact of animal body size and shape on camouflage and identify research issues and challenges. A negative correlation between background matching effectiveness and an animal's body size has been reported, whereas flatter body shapes enhance background matching. The effectiveness of disruptive coloration is also negatively correlated with body size, whereas irregular body shapes physically disrupt the body outline, reducing the visibility of true edges and making it challenging for predators to identify prey. Countershading is most likely in larger mammals with smaller individuals, whereas body size is unrelated to countershading in small‐bodied taxa. Body shape influences a body reflectance, affecting the form of countershading coloration exhibited by animals. Animals employing masquerade achieve camouflage by resembling inanimate objects in their habitats in terms of body size and shape. Empirical and theoretical research has found that body size affects camouflage strategies by determining key aspects of an animal's appearance and predation risk and that body shape plays a role in the form and effectiveness of camouflage coloration. However, the mechanisms underlying these adaptations remain elusive, and a relative dearth of research on other camouflage strategies. We underscore the necessity for additional research to investigate the interplay between animal morphology and camouflage strategies and their coevolutionary development, and we recommend directions for future research.

## Graphical abstract

In this review, we analyzed the research on the impact of animal body size and shape on their camouflage and outlined existing research issues and challenges. Overall, we found a negative correlation between body size and the effectiveness of disruptive coloration and background matching as camouflage strategies. Further, different body shapes also affect countershading effectiveness. Animals employing masquerade achieve camouflage by resembling inanimate objects in their habitats.

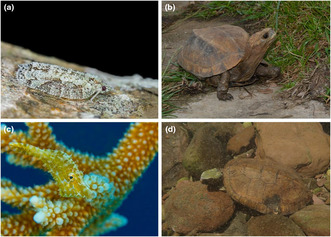

## 1 INTRODUCTION

Camouflage involves a spectrum of strategies that hinder animal detection and recognition, primarily encompassing crypsis, masquerade, and motion dazzle (Stevens & Merilaita, 2009a ). Crypsis includes various sub‐strategies such as background matching, disruptive coloration, and countershading (Johnsen et al., 2004; Stevens & Merilaita, 2009a). Besides the cognitive constraints of the receivers, the success of visually based camouflage relies greatly on the interplay between an animal's appearance and its environment (Skelhorn & Rowe, 2016; Troscianko et al., 2009). Since Erasmus Darwin (1794) first explained the intricate color designs of animals through crypsis, research on animal camouflage has primarily focused on the mechanisms of coloration, brightness, texture, and patterns of body color (Merilaita et al., 2017). However, animal body size and shape are also crucial external features (Stevens & Merilaita, 2011), which often co‐evolve between predators and prey (Carbone et al., 2011). Nonetheless, research on the impact of body size and shape on camouflage has been long overlooked.

Over the past two decades, a gradual increase in focus on the impact of body size and shape on camouflage has led to experimental studies. For instance, Cuadrado et al. (2001) reported that body size affects background matching. King et al. (2023) discovered that the surface‐disruptive coloration effects in three‐dimensional (3D) animal models was more pronounced than that of two‐dimensional (2D) models. Exploring the relationships among body size, body shape, and camouflage is crucial for understanding the adaptation and evolution of camouflage within a broader comparative framework. Therefore, herein, we review the impact of animal body size and shape on camouflage, providing a reference for animal camouflage studies and encouraging further in‐depth exploration of this field. In addition, we highlight future research directions based on existing issues and challenges in the field.

## 2 ROLE OF BODY SIZE IN CAMOUFLAGE

Body size refers to the proportions between different body parts, generally encompassing the size and distribution of body mass and the lengths of various body parts (Clauss et al., 2013; Maher et al., 2022; Peters, 1986). Body size is a fundamental parameter in the life history of animals and is a crucial characteristic for predators during initial target identification (Hall et al., 2013; Stearns, 1992). Animals of different body sizes face varying predation pressures and environmental complexities (Remmel & Tammaru, 2009; Tsai et al., 2016), and body size strongly influences an object's fundamental detection probability (Mänd et al., 2007). Therefore, body size can influence the selection and effectiveness of camouflage strategies.

### 2.1 Body size and crypsis

#### 2.1.1 Body size and background matching

Background matching is a highly prevalent cryptic coloration that reduces an animal's detectability by minimizing visual deviations between the animal's appearance and its background (Stevens & Merilaita, 2009a, 2009b). Phylogenetic studies have indicated that smaller species more often exhibit body coloration that matches their background, as demonstrated by taxa in the superfamily Majoidea and order Artiodactyla, including pigs (Suidae) and peccaries (Tayassuidae) (Caro et al., 2018; Hultgren & Stachowicz, 2009; Stoner et al., 2003). Barnett et al. (2023), on investigating the developmental process of the Fowler's toad *Anaxyrus fowleri* with a lifelong unchanging spotted pattern, found that background matching is most effective in the early stages of development for smaller individuals. Consequently, phylogenetic and ontogenetic studies have indicated a negative correlation between body size and background matching.

Body size plays a pivotal role in predator detection. Large individuals are more prone to detection even when their body pattern remains constant. This increased detectability in larger individuals is attributed to larger non‐matching areas and longer pattern edges against the background (Pembury Smith & Ruxton, 2021; Postema, 2022; Remmel & Tammaru, 2009). Background matching operates primarily by extending the average search time of predators, thereby reducing the probability of prey discovery and serving as camouflage (Viana et al., 2022). In a virtual human predation experiment, the time required to detect individuals of varying body sizes and color morphs of the pygmy grasshopper *Tetrix subulata* in their natural habitats was investigated. The results indicated that irrespective of color morphs, the search time increased with decreasing body size (Karpestam et al., 2014). Similarly, a study involving exposing artificially created larvae of varying sizes, color‐matched to their backgrounds, to avian predators revealed that the probability of avian detection increased with the size of the individual (Remmel & Tammaru, 2009). Furthermore, smaller individuals can effectively conceal most of their bodies in matching environmental fragments within heterogeneous backgrounds, making detection more challenging. For instance, in Fowler's toad, juvenile individuals exhibit a spotted pattern on their bodies that closely matches sand grains; they are also more adept at concealing their bodies beneath larger fragments, such as fallen leaves or other debris in heterogeneous backgrounds. Consequently, detecting juveniles is more challenging than detecting larger individuals in such environments (Barnett et al., 2023).

The significant predation pressure faced by small individuals in the early stages of development considerably influences the choice of animal background matching (Mänd et al., 2007). The insular land crab *Johngarthia lagostoma* exhibits notable intraspecific color variation during development. One study found that only small individuals (carapace width <30 mm) display a black body coloration matching the substrate that reduces the risks of intense and intraspecific predation (João et al., 2023). In contrast, larger individuals exhibit noncamouflaged yellow or purple phenotypes as they face lower predation pressures and have evolved multiple defense strategies (João et al., 2023; Liao et al., 2022; Rojas & Burdfield‐Steel, 2022). Notably, animals often employ a singular defense strategy during the early stages of development when they are more vulnerable to predation. However, this period represents the phase with the highest potential for future reproduction. Consequently, selection pressure favoring background matching is likely to be strongest during the early developmental stages of animals (Berger et al., 2006).

In summary, body size considerably impacts an animal's ability to blend into its environment, as it largely determines the extent to which the animal's appearance differs from its surroundings in terms of patterns and edges. Typically, individuals with a smaller body size demonstrate more effective background matching. Conversely, larger individuals, benefiting from enhanced defensive and evasive capabilities, exhibit reduced reliance on background matching. Notably, the impact of body size on background matching is evident across various species during their life stages.

#### 2.1.2 Body size and disruptive coloration

Disruptive coloration, characterized by bright markings, can lead to the creation of false edges and boundaries, hindering predators from detecting or identifying the true outlines and shapes of their prey (Stevens & Merilaita, 2009a, 2009b). Peters (1983) suggested that body size may influence visual appearance, thereby affecting the effectiveness of disruptive coloration. However, experimental research on the effect of body size on disruptive coloration in prey is limited, and comprehensive experimental data supporting this relationship over an extended period remain lacking. Pembury Smith and Ruxton (2021) examined the ability of wild avian predators to detect artificial lepidopteran prey of different sizes exhibiting either uniform or disruptive coloration. Their results revealed that regardless of coloration type, larger individuals were more likely to be easily detected by predators. Furthermore, compared to uniform coloration, disruptive coloration exhibited poorer concealment effects on larger individuals because of the ineffective disruption of the body outline, preventing them from achieving the desired concealment effect.

The shore crab *Carcinus maenas* is an ideal experimental subject for studying the transformation of disruptive coloration with ontogeny. The polymorphic disruptive coloration patterns in the carapace of juvenile shore crabs diminish with age (Crothers, 1968; Stevens et al., 2014; Troscianko et al., 2021). Nokelainen et al. (2019) observed that irrespective of the background treatment, adult shore crabs consistently adopted a deep green cryptic phenotype. This suggests that disruptive coloration may be an ineffective concealment strategy for individuals with larger body sizes and increased mobility across various backgrounds.

The degree of disruptive coloration decreases with increased body size, which aligns with predator perception and energy allocation in animals. Larger individuals exhibit greater mobility and an increased mismatch between disruptive coloration patterns and the environment, leading to heightened detectability (Nokelainen et al., 2019). Moreover, larger individuals invest more energy in reproduction and alternative defense strategies, gaining enhanced resistance and counterattack capabilities, thereby reducing resource allocation to maintain protective coloration (Ahl et al., 1996; Anderson et al., 2013).

#### 2.1.3 Body size and countershading

Countershading refers to the phenomenon in which an animal's dorsal coloration is darker than its ventral coloration (Thayer, 1909). Terrestrial and aquatic animals employ different mechanisms to protect themselves from shade. Terrestrial animals commonly use self‐shadow concealment to counteract shadows and outline cues caused by illumination, enhancing visual blending with the background (Caro & Koneru, 2021). Self‐shadow concealment is achieved by gradual pigment deposition along the dorsoventral axis of the body, which precisely balances the radiance gradient between the dorsal and ventral regions. When observed from a lateral perspective, the radiance of the entire animal body matches that of the background irradiance, thereby intensifying the degree of background matching and reducing the risk of predator detection (Caro, 2013; Cott, 1940; Rowland, 2009). Using a phylogenetic approach, a study analyzing the dorsal–ventral brightness ratio, which serves as an indicator of countershading, across 63 species of primates, revealed that smaller species tend to exhibit a higher degree of countershading (Kamilar, 2009). Subsequently, Caro et al. (2021) further refined color scoring across different body parts of primates by conducting a phylogenetic comparison and reported similar conclusions. In a pioneering study, Allen et al. (2012) quantified countershading in ruminants. They measured the pixel values from the dorsal to the ventral side in specimen photographs and converted them to reflectance using a camera response function curve. Phylogenetic methods were employed to explore the factors influencing countershading features. This study found a consistent negative correlation between body size and the degree of countershading.

Compared with terrestrial animals, aquatic animals face the challenge of being detected from any angle in the water, prompting the evolution of countershading adaptations that may be consistently viewed against different backgrounds from above and below (Kelley et al., 2017; Kelley & Merilaita, 2015; Wallace, 1889). Furthermore, this adaptive strategy is more widespread than self‐shadow concealment in aquatic animals. A comparative phylogenetic analysis of cetaceans revealed that smaller individuals weighing <500 kg and measuring <5 m exhibited a higher degree of countershading than larger individuals, indicating a negative correlation between countershading and body size (Caro et al., 2011). Smaller mammals face higher predation pressures (Stankowich et al., 2014), potentially driving the evolution of countershading in smaller individuals. Thus, for large‐bodied terrestrial and aquatic mammals, the degree of countershading and body size are negatively correlated, potentially driven by the greater predation risk faced by smaller individuals.

In taxa with smaller individuals and countershading adaptations, for example, flying animals, body size is not the primary factor influencing countershading. Notably, no correlation was found between body size and the degree of countershading in a comparative study of fur coloration patterns and ecological factors in the order Chiroptera (bats) (Santana et al., 2011). The author suggested that this may vary among different species and with lighting conditions, warranting further exploration. In studies on the adaptive functionality of countershading in Procellariiform seabirds, body size has not been found to be a factor influencing countershading (Bretagnolle, 1993). Phylogenetic analyses have similarly indicated no relationship between body weight and countershading in seabirds (Rogalla et al., 2021). The prevailing belief is that the evolution of countershading in seabirds is correlated with changes in light environments (Delhey et al., 2023; Gomez & Théry, 2007).

Lepidopteran larvae also commonly exhibit countershading coloration. In these larvae, larger individuals face a higher predation risk, linked to the disruption of concealment effects and the energy balance of predators (Pembury Smith & Ruxton, 2021; Remmel & Tammaru, 2009). Accordingly, Hwang et al. (2023) predicted that larger lepidopteran larvae may be associated with stronger countershading abilities. However, phylogenetic analyses of larvae in the families Erebidae, Geometridae, Saturniidae, and Sphingidae, and field predation experiments with artificial prey indicated a correlation between larger individuals and countershading only in Saturniidae. This association was primarily due to a higher proportion of species in Saturniidae with leaf‐like shapes, suggesting that the degree of countershading may not be strongly correlated with body size, but rather with the species' preferences for light environments based on their body shape (see below). Additionally, comparative studies investigating the relationship between ecological/morphological variables and the degree of countershading in Sciuromorpha revealed an association between light environments and countershading (Ancillotto & Mori, 2017). Therefore, for taxa with smaller individuals, body size may not be a factor influencing the degree of countershading, but rather the light environment.

#### 2.1.4 Summary

Body size is negatively correlated with the effectiveness of background matching, disruptive coloration, and countershading. Although the relationship between countershading and body size has only been confirmed in large mammals, smaller animals preferably use the crypsis strategy. This preference could be primarily because smaller animals may face higher predation pressure and exhibit weaker secondary defense capabilities. Consequently, crypsis strategies offer the most effective protection for them. Furthermore, the visual concealment of prey depends on the interaction between the detectability of an individual's physical appearance and their adaptability to their complex environment. In conclusion, smaller animals more effectively use crypsis strategies than larger individuals.

### 2.2 Body size and masquerade

Masquerading is an evolutionary adaptation in which animals resemble inedible and often inanimate objects in their habitats, such as rocks, bird droppings, fallen branches, or leaves, leading to misidentification by predators and causing them to abandon their pursuit of the prey (Skelhorn, Rowland, & Ruxton, 2010). Masquerade can be categorized into two types: element and object imitation. In element imitation, animals resemble specific and common elements of the environment, such as dried leaves or tree branches, and the masquerading species must be situated in the same background as the object being imitated. Conversely, animals resemble uncommon environmental elements in object imitation, such as bird droppings (Mark et al., 2021; Skelhorn, Rowland, & Ruxton, 2010).

Element imitation requires matching the size of the object being imitated to maximize masquerading benefits. Skelhorn, Rowland, Speed, et al. (2010) and Skelhorn and Ruxton (2011) conducted experiments using the domestic chicken *Gallus gallus domesticus* as a predator and assessed the masquerade effectiveness of early thorn moth *Selenia dentaria* caterpillars mimicking twigs on branches of various lengths and densities. The results revealed that predators took longer to detect the larvae when the larvae were similar in length to the branches, or when the branch density was high. Further research on the habitat preferences of larvae revealed that smaller individuals preferentially chose smaller branches. In contrast, larger larvae preferred larger branches, suggesting that element imitation is subject to selective pressure based on body size (Skelhorn & Ruxton, 2013). Additionally, Folgar‐Cameán et al. (2022) discovered that flea beetles (Chrysomelidae: Alticini) achieve masquerade protection by hiding among their own feeding damage spots similar in size to their body. Interestingly, larger individuals leave larger and deeper feeding holes on leaves, which better matches the size and depth of the feeding damage. A computer‐simulated predation experiment indicated that as the number of feeding holes increased and the hole size approached the body size of the beetles, the visual search efficiency of the human observers decreased (Ren et al., 2018). This provided flea beetles with more time to jump or roll away from the leaf surface. Notably, the animal must match the background environment of the imitated object to benefit from element imitation (Mark et al., 2021). If the masquerading animal does not match the size of the imitated object, then predators or prey can easily identify the masquerader based on perception and experience. Therefore, element imitation enforces strict limitations based on the body size of animals. Masqueraders of different sizes choose imitated objects that match their size to achieve maximum protective effects.

Animals engaged in object imitation experience less selective pressure based on body size because objects such as stones or bird droppings are commonly found in various microhabitats. This allows an animal engaging in object imitation to be mistakenly identified as a model in a broader range of backgrounds (Skelhorn, Rowland, & Ruxton, 2010). However, masquerade becomes ineffective when the size of the masquerading animal does not match the predator's experience of the model's size (Skelhorn, Rowland, & Ruxton, 2010). Some young lepidopteron larvae that adopt bird‐dropping masquerades alter their defensive strategies at later stages because the larger size exceeds a predator's or prey's expectation based on their experience of the actual size of bird droppings. Additionally, the masquerade effect is prone to disruption owing to the frequent movements associated with animal activities, such as feeding and pupation (Futahashi & Fujiwara, 2008; Gaitonde et al., 2018; Postema, 2022; Valkonen et al., 2014). Yu et al. (2022) explored ontogenetic changes in defensive coloration in the bird‐dropping crab spider *Phrynarachne ceylonica*. They found that females gradually transitioned from crypsis to bird‐dropping masquerade starting from the fourth instar. This shift was attributed to the small size of juvenile spiders, ranging from 1 to 2 mm, which is much smaller than real bird droppings. Additionally, Stückler et al. (2023) reported that adult Wallace's flying frogs, *Rhacophorus nigropalmatus*, exhibit green dorsal coloration, whereas juveniles display a bright red coloration; however, 1 month after metamorphosis, white spots appear on their backs. The authors hypothesized that juveniles adopt the bird‐dropping masquerade strategy. However, the results of field predation experiments showed that while the white spots reduced the likelihood of predation, protection through bird‐dropping masquerade was unlikely. A more plausible explanation is that the white spots, in combination with the bright red body coloration, produced an aposematic effect because the size of the artificial models used in the experiments may have exceeded the experimental range for bird droppings. Therefore, although animals engaged in object imitation are not strictly limited by body size, a considerable disparity in size between the masquerader and the object can affect its effectiveness.

The effectiveness of a masquerade depends primarily on the prey's recognizability and the predator's experience. Therefore, body size has different effects on the two masquerade strategies. A match between body size and the imitated object is crucial for element imitation, whereas object imitation experiences relatively less selective pressure based on body size and can provide protection across a broader size range. These findings reveal the adaptability of animals in masquerade, providing valuable information for a deeper understanding of the effectiveness of this camouflage strategy.

## 3 ROLE OF BODY SHAPE IN CAMOUFLAGE

Body shape refers to the geometric characteristics of an animal's body, encompassing both 2D and 3D shapes. Shape represents the global geometric property of an individual that remains unaffected by rigid motions or overall size scaling. In computer vision and model recognition, shape serves as a 2D representation of a region of interest and can be perceived as the outline of the target (Paquet et al., 2000; Stevens et al., 2011). Identifying shapes and outlines is crucial for object recognition (Merilaita et al., 2017). To better perceive cues related to the shape and outline of prey, and to discern differences between camouflaged prey and matching targets, some predators have evolved heightened sensitivity or stereovision (Barnett et al., 2018; Galloway et al., 2020). Simultaneously, in the dynamic processes of evolution and adaptation, animals can enhance their camouflage effectiveness through the intrinsic features of their body shape.

### 3.1 Body shape and crypsis

#### 3.1.1 Body shape and background matching

The body shape of prey plays a pivotal role in the delicate balance between background matching and detection risk (Cammack, 2017; Schmitz, 2017). Background matching enables a prey animal's appearance to harmonize with the color, brightness, and patterns of one (specialized) or multiple (generalist) background types, thereby reducing its visual detection by predators (Stevens & Merilaita, 2009a). In contrast, the body shape or outline of the prey provides numerous additional cues, such as shadows and depth information. Notably, the discontinuity between an animal's body and its background generates distinct visual clues even with backgrounds with high‐fidelity color and texture matching, facilitating predator detection and identification (Thayer, 1918). Furthermore, the evolution of binocular vision and the ability to detect depth and 3D observation of the surroundings increased a predator's ability to detect the shape and presence of prey (Heesy, 2009; Julesz, 1971).

**FIGURE 1:**
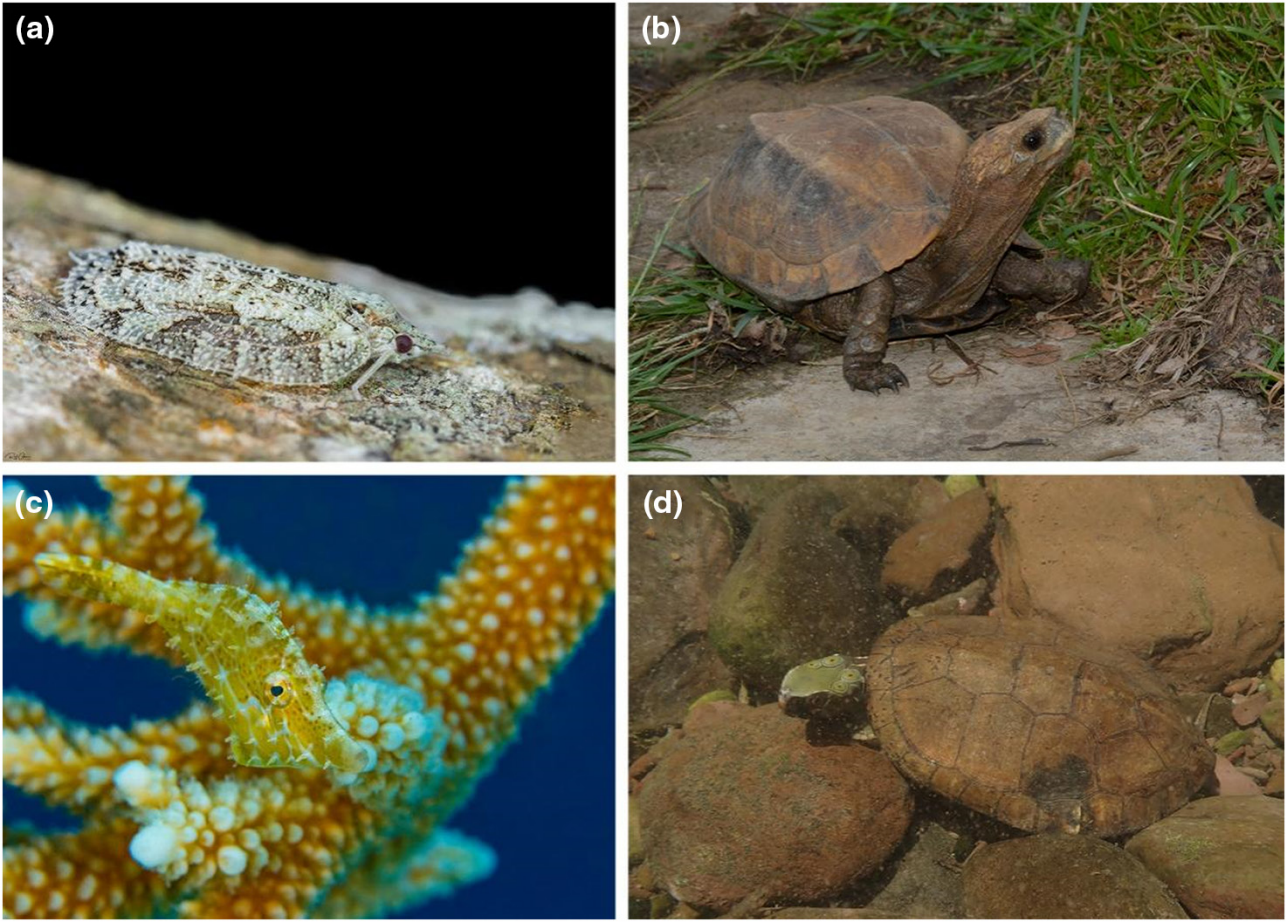
Examples of the impact of body shape on camouflage. (a) A flatid hopper (Flatidae) with flatoidinisation syndrome, which enhances background matching; image: Rafi Amar. (b) A keeled box turtle (*Cuora mouhotii*) lacking conspicuous disruptive coloration but with a distinctive trapezoidal body shape that creates a shape‐disruptive camouflage effect; image: Fanrong Xiao. (c) A slender filefish (*Monacanthus tuckeri*) with dermal flaps exhibiting an irregular marginal form; image: Douglas Klug. (d) Four‐eyed turtle (*Sacalia quadriocellata*) masquerading as a stone; image: Fanrong Xiao.

Using binocular vision in predators to disrupt camouflage has led to the evolution of counter‐adaptive features in prey that protect from the depth perception of binocular vision. This adaptive response is advantageous for prey in the coevolutionary arms race. Mid‐cretaceous amber fossils revealed that numerous hemipteran groups employed flatoidinisation syndrome, combining body shape, color, and behavior to create intricate camouflage (O'Brien, 2002; Szwedo & Stroiński, 2017). Members of the planthopper family Flatidae, especially the subfamily Flatoidinae (Hemiptera: Fulgoroidea), employ dorsoventral flattening to eliminate actual or perceived shadows and minimize outline cues (Figure 1a). Their flat shape and coloration make them less detectable on tree bark, lichens, or other plant surfaces. Lateral wings or irregular body projections often blend with the bark, bridging the gap between the body and background matrix. These features simulate tree trunk textures and enhance background matching (He et al., 2022; Jiang et al., 2019). However, direct experimental support for the efficacy of body‐flattening features in background matching is lacking. Therefore, whether prey can enhance their survival chances by adjusting their body shape to improve background matching requires further experimental validation.

#### 3.1.2 Body shape and disruptive coloration

Cryptic prey employ disruptive coloration and leverage high‐contrast patterns on their body surfaces to disrupt body outlines or real edges (Cuthill et al., 2005), suggesting a relationship between body shape and disruptive coloration (Merilaita & Lind, 2005). However, studies on the direct influence of body shape on disruptive coloration are scarce. Bu et al. (2020) revealed that the distinctive body shape of the keeled box turtle *Cuora mouhotii* generates shape disruption effects, surpassing the effects produced by conspicuously colored stripes (Figure 1b). The flattened and trapezoidal shape of the turtle's dorsal shell, with steep sides and a wider bottom, creates two distinct parallel planes, resulting in a bright difference between the central and lateral stripes owing to depth variation. This difference in brightness creates a false edge, making the internal edge more noticeable to predators with binocular vision while concealing the real edge connected to the background environment. This effect resembles surface‐disruptive camouflage and is generated by body shape rather than body color. King et al. (2023) conducted field experiments to ascertain the effectiveness of 3D artificial prey with physically disrupted surfaces against predation. They found that this prey had a survival advantage over continuous‐surface 3D prey and 2D prey with edge‐enhanced disruptive camouflage. This superiority is attributed to strong perceptual segregation cues arising from depth variation on the 3D surface, causing erroneous boundary resolution by avian predators and, consequently, misidentification of the target. Therefore, body shape can lead to disruptive camouflage effects.

The body shape of animals is associated with the orientation of stripes. Many fish species exhibit eye stripes, which have been demonstrated to serve a disruptive coloration function through differential blending (Stevens & Merilaita,  2009b), concealing the noticeable shape of the eye (Kjernsmo et al., 2016). Barlow (1972) observed that longitudinal eye stripes are frequently found in slender fish species, whereas vertical stripes tend to occur in deep‐bodied fish. Specific color patterns and the slender body shape of animals can synergistically enhance disruptive camouflage (Castillo & Tavera, 2022). For example, the blue‐spotted cornetfish *Fistularia commersonii*, which lives in coral reefs, exhibits a longitudinal striped pattern distributed across its entire body. Similar to the eye stripes, these stripes contribute to disruptive camouflage through differential blending. The striped pattern on this fish partially matches the color of coral reefs, which disrupts the body outline, thereby facilitating disruptive coloration. When the fish are stationary, close to the sea bottom, and the disruptive pattern is not evident in a complex background (such as coral reefs with many branches), the disruptive ratio using the GabRat method (Troscianko et al., 2017) exceeds 0.2, suggesting that the unique elongated shape of blue‐spotted cornetfish contributes to its subtle disruptive effects. However, the disruptive ratio exceeds 0.4 when adopting disruptive patterns, indicating highly effective disruptive camouflage. Notably, detecting the body outline is challenging for predators. Therefore, combining the slender shape of the blue‐spotted cornetfish with disruptive patterns improves the disruptive camouflage effect, significantly reducing its detectability (Castillo & Tavera, 2022).

Beyond the 3D body shape of animals influencing disruptive coloration, Cott (1940) proposed that structural or morphological modifications of the animal's outline can obscure the shape, thereby increasing the effectiveness of disruptive camouflage. This phenomenon is known as irregular marginal form. The comma butterfly *Polygonia c‐album* with its irregular and intricate wing outlines, is a typical example (Stevens & Merilaita,  2009b). Research in visual science and psychology has indicated that highly curved outlines reduce the salience of edge information, impairing shape processing (Panis & Wagemans, 2009). Folded outlines on the body are more effective for concealment than are straight boundaries (Webster et al., 2015), as straight boundaries are detected by vertebrates more efficiently than curved ones (Bell et al., 2010). Similarly, 3D protrusions on the body have similar effects. The slender filefish *Monacanthus tuckeri* (Figure 1c), a coral reef species capable of rapidly changing its body pattern based on the surrounding environment, possesses numerous small and protruding dermal flaps on its body. These dermal flaps can create false edges on the body surface, enhancing disruptive camouflage (Allen et al., 2015). Rohr et al. (2021) discovered that prominent, newly grown feathers on the young snowy plover *Charadrius nivosus* also reduce edge strength. However, experiments to determine whether body protrusions can decrease detection by predators are lacking. Measuring detection times or survival probabilities with or without similar protrusions will help evaluate this mechanism's real‐time relevance.

In summary, 3D body shapes with depth variation facilitate disruptive camouflage by eliciting perceptual segregation cues in predators, which does not necessarily rely on body color. Furthermore, the orientation of eye stripes in fish, which contribute to disruptive camouflage, is correlated with body shape. Specifically, longitudinal eye stripes are frequently observed in slender fish species, while vertical stripes tend to occur in deep‐bodied fish. In some animals that exhibit disruptive coloration, slender body shapes can enhance the disruptive effect, facilitating integration with heterogenous backgrounds. Additionally, animals can further enhance disruptive camouflage by employing irregular edge forms to reduce the salience of edge information, and protrusions on edge outlines have a similar effect. Hence, combinations of 3D body shape, slender body shape, irregular edges, and protrusions contribute to effective disruptive camouflage, showcasing the diverse strategies employed by animals for concealment.

#### 3.1.3 Body shape and countershading

Countershading, achieved through increased background matching or reduced shape cues, is a mechanism of visual deception. Although optimal countershading is influenced by body shape (Penacchio et al., 2015, 2018), it remains understudied. Harris et al. (2016) demonstrated evolutionary adaptation in prey by measuring the reflectance and body shape of various lepidopteran larvae, suggesting that body shape matching with reflectance is a crucial adaptation. This provides a method for assessing the impact of different body shapes on countershading effects. Penacchio et al. (2021) investigated the influence of body shape and color on countershading effects using 3D modeling to explore the interactions between shape and reflectance in the larvae of six different moth species (three exhibiting countershading). By exchanging their shapes and reflectance, they determined the visibility of the resulting “hybrid bodies” under different ecologically relevant lighting conditions. Notably, individuals with countershading that adopted the shapes of other species exhibited significantly increased visibility, suggesting a coordinated shape and reflectance adaptation to minimize visibility to predators. Hwang et al. (2023) evaluated the potential association between countershading and body size in lepidopteran larvae employing comparative phylogenetic analysis with field predation experiments. Surprisingly, no significant correlation was observed between countershading and body size; however, the evolution of countershading in species with leaf‐like body shapes was reported. Twig‐resembling species may rely more on masquerade for protection. Leaf‐resembling species, that typically inhabit leaves, are more exposed to light from the sky and may be affected by the light conditions in species‐specific habitats (Allen et al., 2012). Consequently, the pressure to eliminate the vertical luminance gradient may be stronger in leaf‐resembling species than in twig‐resembling species (Cuthill et al., 2016). For instance, the polymorphic caterpillar of the fig sphynx *Pachylia ficus* can assume either twig‐ or leaf‐resembling forms, but only the leaf‐like form exhibits countershading (Hwang et al., 2023). Future research should focus on a more in‐depth exploration of specific body shape features and their relationship with reverse camouflage to comprehensively understand this phenomenon.

Aquatic organisms with different body shapes employ distinct countershading mechanisms to achieve effective concealment. Countershading in aquatic fauna manifests as a gradient of color from the dorsal to the ventral sides and a sharp division between dark and light coloration near the midline on the flank, representing two different countershading mechanisms: self‐shadow concealment and dorsoventral background matching (Caro et al., 2011). For instance, the Atlantic hump‐backed dolphin *Sousa teuszii*, with prominent long ridges on its back, adopts gradient countershading, whereas the more slender‐bodied Atlantic white‐sided dolphin *Lagenorhynchus acutus* displays sharp countershading (Caro et al., 2011). This suggests that dorsally raised aquatic animals may favor self‐shadow concealment, although further research is needed to confirm this hypothesis. Investigations regarding whether aquatic animals utilize self‐shadow concealment or background matching using the rainbowfish *Melanotaenia australis* as a model have revealed that fish are more likely to achieve concealment through background matching than through self‐shadow concealment (Kelley et al., 2017; Kelley & Merilaita, 2015). Similarly, many deep‐sea sharks employ counter‐illumination through ventral bioluminescence to better match the brighter background radiance from above, concealing their outlines (Claes et al., 2010). This suggests that the body shape of fish may be better suited for utilizing background‐matching mechanisms for countershading. However, the correlation with specific body shape features requires further exploration. Sea turtles represent a unique example, employing both self‐shadow concealment and dorsoventral background matching (Bolten et al., 2003; Bustard, 1970). The raised dorsal ridge and flattened ventral side of sea turtles may contribute to dual countershading mechanisms, allowing them to efficiently evade predators from multiple angles (Ryan et al., 2022; Salmon et al., 2016).

Current research indicates a complex interaction between body shape and reflectance, which influence the visibility of countershading species across diverse backgrounds, affecting the selection of countershading mechanisms in animals. Therefore, body shape may be a crucial ecological factor affecting countershading. However, in‐depth research on the specific relationships between body shape features and countershading remains lacking. Notably, countershading is a complex adaptive strategy involving multiple body shape and color factors. Therefore, the impact of body shape features on countershading should be systematically compared and analyzed to explore their potential evolution and adaptive significance, establishing a more comprehensive and precise theoretical framework for understanding countershading camouflage.

### 3.2 Body shape and masquerade

Animal body shape plays a pivotal role in influencing masquerade, as different species have evolved distinct morphological features based on their survival environments and anti‐predatory needs to enhance object matching and reduce recognizability. For instance, the four‐eyed turtle *Sacalia quadriocellata* chooses habitats in rivers with stones resembling its body shape, with individual masquerade efficiency influenced by the similarity between individuals and imitated objects (Figure 1d). Xiao et al. (2021) conducted a pioneering quantitative assessment of the masquerade efficiency of the four‐eyed turtle using morphological descriptions. Notably, midstream individuals showed a significantly higher shape similarity to river stones than those upstream and downstream, with predator simulation experiments indicating the highest masquerade efficiency in midstream individuals. This suggests a positive correlation between the shape similarity of the four‐eyed turtle and river stones and masquerade efficiency. Additionally, the keeled box turtle, resembling an inverted trapezoid when half‐buried in fallen leaves, effectively reduces its recognizability by closely matching the shape and color of leaves in the leaf litter (Bu et al., 2022). Hence, animals benefit from masquerade when their overall or partial body shapes resemble those of their models.

Although most camouflage patterns in nature are static, cephalopods, especially squid and octopus, exhibit dynamic camouflage through skin tissues capable of surface deformation. Initially, these cephalopods visually process substrates, such as rough rocks or corals, and then manipulate the 3D physical texture of their skin to closely resemble their surroundings. The reversible transformation from smooth to uneven textures further enhances the similarity of squid and octopus to various objects in different environments (Panetta et al., 2017). Subsequently, scientists have developed stretchable surfaces with programmable materials that can alter appearance and texture in response to external environmental changes (Pikul et al., 2017).

In element imitation, the body size and shape consistency between an individual and the imitated object are paramount to optimal effectiveness. Body size and shape uniformity contribute significantly to achieving a more efficient masquerade effect in specific backgrounds (Mark et al., 2021). Notably, discrepancies in body size are tolerable; however, body shape should be closely matched in object imitation. This implies that an animal's body shape should represent a scaled or adjusted version of the object being mimicked to ensure visual congruence rather than an evident mismatch. For example, suppose an animal masquerades as a round object such as a pebble, as seen in the case of the four‐eyed turtle masquerading as a river stone. In that case, its body shape should exhibit corresponding angles and edges, rather than being square or of other shapes, maintaining visual consistency with the imitated object (Xiao et al., 2021).

The resemblance between the animal body shape and the model is crucial for effective masquerade, involving either the overall body shape or specific body parts. Matching or resembling different models can confer anti‐predatory benefits. Despite research having been carried out to determine these aspects, in‐depth empirical research on the relationship between animal body shape and masquerade effects, especially concerning classic examples such as the dead leaf butterfly *Kallima inachus*, is required to clearly identify the mechanisms by which animal body shape promotes masquerade evolution.

## 4 EXISTING ISSUES AND FUTURE RESEARCH

### 4.1 Disparities in research progress on various camouflage strategies

Current research on the associations among body size, body shape, and camouflage primarily focuses on camouflage types such as background matching, disruptive coloration, countershading, and masquerade. The relationships among body size, body shape, and other camouflage types, such as flicker‐fusion camouflage (Umeton et al., 2017) and distractive markings (Merilaita et al., 2013), remain unexplored. Understanding the correlations between these camouflage types and individual characteristics will further our understanding of how animals adapt to their environments. There is a significant disparity in the research progress on various camouflage strategies. Therefore, future research should include systematic comparative phylogenetic studies and empirical investigations across a broad range of animal taxa.

We acknowledge that there is still controversy over whether the motion dazzle strategy can conceal or mislead signals of animal movement (Tan et al., 2024), although motion dazzle was initially considered a camouflage method to conceal prey movement (Stevens & Merilaita, 2009a). Therefore, we do not consider motion dazzle as a camouflage strategy, and so, it was not included in our review (Scott‐Samuel et al., 2023).

### 4.2 Knowledge gap regarding the relationship between camouflage and body shape

Independent experiments are required to clearly understand the effects of body shape on prey detection and camouflage effectiveness. The dynamic evolution of camouflage in relation to body shape is highly complex and influenced by the visual perception of observers (Galloway et al., 2020). This underscores the need for further research that considers the visual capabilities of observers, considering consistency and variation across different species, environments, and visual systems, while controlling for other factors such as body color, hue, and motion. Most studies focus on a single species or background and lack sufficient generalizability and comparability. For instance, the Indochinese box turtle *Cuora galbinifrons* exhibits typical disruptive coloration. However, Bu et al. (2020) found that the GabRat value for the keeled box turtle, which lacks disruptive coloration, is significantly higher than that for the Indochinese box turtle. This difference may be related to the unique body shape of the keeled box turtle. However, Bu et al. (2020) measured only GabRat values without testing their anti‐predation efficiency or different body shape indicators to determine their influence on GabRat values. Experimental and mathematical modeling approaches should be employed in future studies to assess the impact of body shape features on camouflage effectiveness, contributing to the enrichment of theories related to animal camouflage.

### 4.3 Lack of research on body size and body shape in 3D camouflage

In addition to 2D camouflage strategies, such as background matching and countershading, animals can employ 3D camouflage to disrupt the perception of predators of their 3D structure and depth information. 3D camouflage utilizes the illusion of 3D structure created by patterns, textures, or colors on the surface of an object to generate false edges or shadows. This strategy can make the prey appear larger or smaller than the actual size or blend with the background (Kelley et al., 2022). For example, the European cuttlefish *Sepia officinalis* alters its body color pattern to create depth cues or illusionary depths resembling 3D objects in the background through 3D background matrix and shadows (El Nagar et al., 2021). The effectiveness of 3D camouflage depends on the prey's characteristics, such as body shape, and on the predator's visual system, particularly whether they have binocular vision. Binocular vision processes depth information, allowing predators to distinguish between the prey and the background. However, predators can be deceived using 3D camouflage. Hence, differently shaped prey may employ distinct 3D camouflage strategies and exhibit different effects when adapting to various predators and environments. However, whether prey with different body shapes employ varied 3D camouflage strategies and can maintain these effects at different distances or angles remains unclear. These questions can be answered by using new methods to measure depth perception in animals. Additionally, a deeper understanding of the mechanisms related to predator perception and cognition will enhance our understanding of animal camouflage, as prey defense is ultimately driven by predator selection.

### 4.4 Lack of research on body size and body shape in predator camouflage

Most studies focus on camouflage as a prey adaptation for evading predators, meaning that our understanding of the roles of body size and shape in predator camouflage is limited. Although we consider that some camouflage mechanisms, such as maximizing the reduction in detection and recognition, are universal and thus also apply to predators, predator camouflage differs significantly from that of prey. Predator camouflage aims to avoid detection by not only other predators but also, more importantly, by prey (Pembury Smith & Ruxton, 2020). In fact, the body size and shape of predators determine their energy requirements and hunting range and influence the association between their environment and visual appearance (Pembury Smith & Ruxton, 2020). Even though larger individuals are more conspicuous (Main, 1987), predators must evolve ways to maintain both their large size and camouflage. Therefore, predator camouflage may involve more complex mechanisms and selection pressures, rendering many predatory camouflage strategies unique. However, research on the effects of body size and shape on predator camouflage is currently lacking, particularly in terrestrial ecosystems. This gap imposes limitations and may lead to biases in the development of theories and applications related to animal camouflage.

### 4.5 Lack of research on the interactive effects of body size and shape on camouflage

**FIGURE 2:**
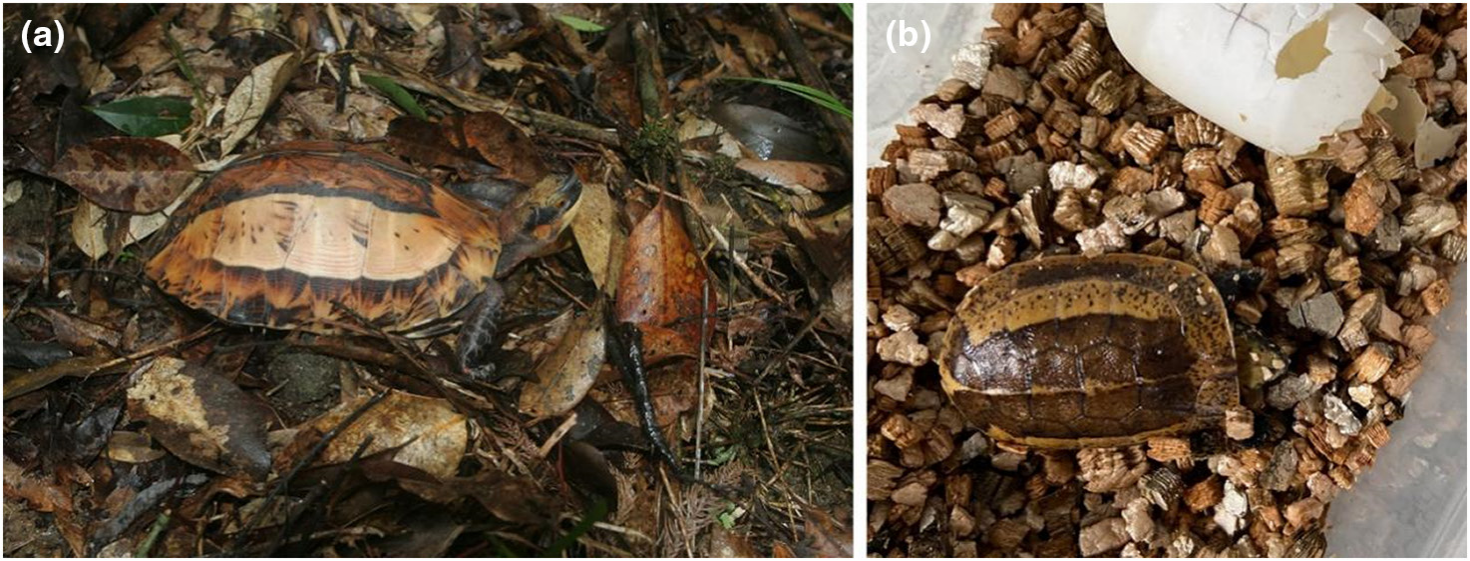
Divergent body shape in adult and juvenile Indochinese box turtles (*Cuora galbinifrons*). (a) Adult Indochinese box turtle featuring a highly arched carapace; image: Fanrong Xiao. (b) Newly hatched Indochinese box turtle with a relatively flat carapace; image: Zhenwang Fa.

Animal body shapes undergo changes during development. For instance, in the developmental process of terrestrial turtles (e.g., Indochinese box turtle), the dorsal shell gradually changes from flat to high‐arched as the individual grows (Figure 2a,b) (Xiao et al., 2023). Therefore, small size and flat shape may contribute to background matching, disruptive coloration, and other camouflage strategies, thereby enhancing camouflage effectiveness. However, being large and high‐arched increases detectability and stereopsis, thereby reducing camouflage effectiveness. Empirical studies on the interactive effects of body size and shape on camouflage are currently lacking.

## 5 CONCLUSION

Camouflage has undergone prolonged coevolution between prey and predators and is a primary function of defense coloration. The forms and mechanisms of animal camouflage are diverse, with body size and shape being two crucial factors that can influence the degree of matching with the background, visibility of edge information, and sensitivity of visual systems. This review summarized theoretical and empirical research findings analyzing the effects of body size and shape on animal camouflage and their ecological and evolutionary significance. We found that the effectiveness of camouflage coloration varies with body size. On one hand, the success of camouflage relies on the cognitive constraints and visual acuity of the receiver (Skelhorn & Rowe, 2016; Troscianko et al., 2009). Larger individuals are more conspicuous (Main, 1987), implying that the maintenance cost and intensity of camouflage coloration may increase, leading to a less pronounced reliance on such coloration (Anderson et al., 2013; Remmel & Tammaru , 2009).

On the other hand, smaller individuals may face higher predation risks and possess weaker defense and resistance capabilities (Berger et al., 2006; Mänd et al., 2007). Consequently, smaller animals tend to rely more on camouflage for protection, using background matching, disruptive coloration, and countershading strategies. Currently, a negative correlation between body size and countershading has only been observed in large mammals. Masquerade camouflage is most beneficial when the body size matches the model size. Body shape is a crucial element that contributes to the detectability and identification of prey. Therefore, some prey species exploit their body shape to enhance camouflage or directly produce camouflaging effects in a coevolutionary arms race with predators. For example, the flat shape of the carapace in the keeled box turtle contributes to disruptive coloration camouflage (Bu et al., 2020).

Understanding the complex and diverse phenomenon that is animal camouflage requires a comprehensive consideration of various factors, including environmental, behavioral, physiological, and genetic elements. Although some aspects of animal camouflage are understood, a gap exists in our understanding of camouflage strategies, with many unanswered questions. These include lacking research on distractive markings, flicker‐fusion camouflage, and 3D camouflage. In addition, the relationship between camouflage and body shape requires further investigation.

In future, more laboratory experiments and comparative analyses are needed to comprehensively explore camouflage strategies. For example, given that camouflage requires interplay between the camouflaged animal and the observer (Skelhorn & Rowe, 2016; Troscianko et al., 2009), empirical studies should be conducted from the perspectives of both prey and predators. Continuous improvement and refinement of methods for morphological quantification, color analysis, camouflage effectiveness quantification, and visual perception are needed. Comparative analyses of camouflage should focus on clades with a wide range of body sizes and unique body shapes to gain a deeper understanding of their evolutionary dynamics and adaptive advantages in natural selection. Interdisciplinary collaborations are essential for providing more comprehensive and in‐depth explanations of the ecological causes of morphological variation in organisms, offering a deeper understanding and a broader perspective on the adoption and functionality of animal camouflage and its relationships with other ecological and evolutionary processes.

## AUTHOR CONTRIBUTIONS

**Hongmin Yu:** Conceptualization (lead); data curation (lead); formal analysis (lead); funding acquisition (lead); investigation (lead); methodology (lead); project administration (lead); resources (lead); supervision (lead); validation (lead); writing – original draft (lead); writing – review and editing (lead). **Zhixue Lin:** Data curation (equal). **Fanrong Xiao:** Conceptualization (equal); writing – review and editing (equal).

## FUNDING INFORMATION

Hainan Provincial Natural Science Foundation of China, Grant/Award Number: 319MS047.

## CONFLICT OF INTEREST STATEMENT

The authors declare no conflicts of interest.

## Data Availability

Data sharing is not applicable to this article as no new data were created or analyzed in this study.
